# Molecular Insights into Reprogramming-Initiation Events Mediated by the OSKM Gene Regulatory Network

**DOI:** 10.1371/journal.pone.0024351

**Published:** 2011-08-31

**Authors:** Nancy Mah, Ying Wang, Mei-Chih Liao, Alessandro Prigione, Justyna Jozefczuk, Björn Lichtner, Katharina Wolfrum, Manuela Haltmeier, Max Flöttmann, Martin Schaefer, Alexander Hahn, Ralf Mrowka, Edda Klipp, Miguel A. Andrade-Navarro, James Adjaye

**Affiliations:** 1 Computational Biology and Data Mining Group, Max Delbrück Center for Molecular Medicine, Berlin, Germany; 2 Max Planck Institute for Molecular Genetics, Molecular Embryology and Aging Group, Dresden, Germany; 3 Genomatix Software GmbH, München, Germany; 4 Theoretical Biophysics, Humboldt University, Berlin, Germany; 5 Experimental Nephrology, Internal Medicine III, Universitätsklinikum Jena, Friedrich-Schiller-Universität Jena, Jena, Germany; 6 The Stem Cell Unit, Department of Anatomy, College of Medicine, King Saud University, Riyadh, Saudi Arabia; Deutsches Krebsforschungszentrum, Germany

## Abstract

Somatic cells can be reprogrammed to induced pluripotent stem cells by over-expression of OCT4, SOX2, KLF4 and c-MYC (OSKM). With the aim of unveiling the early mechanisms underlying the induction of pluripotency, we have analyzed transcriptional profiles at 24, 48 and 72 hours post-transduction of OSKM into human foreskin fibroblasts. Experiments confirmed that upon viral transduction, the immediate response is innate immunity, which induces free radical generation, oxidative DNA damage, p53 activation, senescence, and apoptosis, ultimately leading to a reduction in the reprogramming efficiency. Conversely, nucleofection of OSKM plasmids does not elicit the same cellular stress, suggesting viral response as an early reprogramming roadblock. Additional initiation events include the activation of surface markers associated with pluripotency and the suppression of epithelial-to-mesenchymal transition. Furthermore, reconstruction of an OSKM interaction network highlights intermediate path nodes as candidates for improvement intervention. Overall, the results suggest three strategies to improve reprogramming efficiency employing: 1) anti-inflammatory modulation of innate immune response, 2) pre-selection of cells expressing pluripotency-associated surface antigens, 3) activation of specific interaction paths that amplify the pluripotency signal.

## Introduction

Human embryonic stem (ES) cell research has been fuelled by the potential of using their regenerative properties in cell replacement therapies. To date, only three clinical trials using embryonic stem cell therapy have been approved by the U.S. Food and Drug Administration (FDA) for spinal cord injury patients [1]) and two forms of macular degeneration (ClinicalTrials.gov Identifiers NCT01345006 and NCT01344993).

Scientific, ethical and regulatory issues exclude the widespread use of embryonic stem cells as therapeutic transplantation material. In contrast, induced pluripotent stem (iPS) cells offer advantages over ES cells. iPS cells can be derived from somatic cells, such as fibroblasts, thus bypassing the need for blastocyst-derived ES cells. Furthermore, because iPS cells are derived from the patient's own cells, they are thought to represent a renewable and immunologically compatible cell source for cell replacement therapy, though recent publications have questioned the validity of this general assumption [2], [3], [4], highlighting the need to investigate differences between iPS and ES cells.

Since the landmark discovery that somatic cells can be reprogrammed to an embryonic-like state to create iPS cells by over-expressing a combination of four core transcription factors, consisting of OCT4, SOX2, with either KLF4 and c-MYC (OSKM) or LIN28 and NANOG (OSLN) [5], [6], many variations of the induction protocol have been developed, including the replacement of some of the core factors by others (Nr5a2, Esrrb, Prmt5 [7], [8], [9]) or chemicals (PD0325901, A-83-01, E-616452, AMI-5, kenpaullone [10], [11], [12], [13], [14]), and different methods of delivery into cells, such as non-integrating adenoviruses, episomal-based plasmids, protein delivery, and transfection of *in vitro* generated mRNAs [15], [16], [17], [18].

Despite the abundance of publications on the derivation of iPS cells, we still have a limited knowledge on how the core factors induce pluripotency at the molecular level [17], [19], [20], [21], [22]. To gain insights into this, we profiled transcriptional changes occurring during the early (24, 48 and 72 h post-transduction) stages of reprogramming of somatic human fibroblasts (HFF1), employing the Yamanaka factors (OCT4, SOX2, KLF4 and c-MYC). We observed activated expression of a number of pluripotency-associated genes at these early time points. Finally, we assessed the effect of the reprogramming protocol on reactive oxygen species (ROS) levels, induced DNA damage, activation of p53 and senescence. Based on these findings, we propose three complementary strategies for enhancing the efficiency of reprogramming based on initiating pluripotency amplification pathways, pre-selecting cells expressing pluripotency-associated cell surface antigens, and transiently suppressing innate immune response triggered by the perturbation of cells by the exogenous reprogramming factors.

## Results

### Transcriptional changes accompanying retroviral transduction of the reprogramming factors- OSKM into HFF1 cells

**Figure 1:**
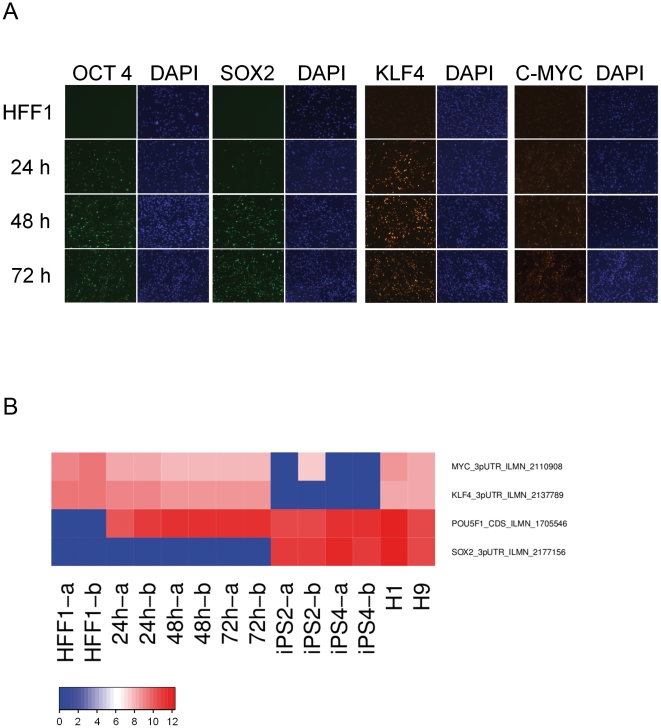
Exogenous OSKM is expressed in transfected HFF1 cells. A. Retrovirus-based expression of OSKM was detected by immunofluorescence at four different time points (HFF1, 24 h, 48 h, 72 h). For reference, nuclear DNA is stained with DAPI (4′,6-diaminidino-2-phenylindole). B. Heat map of microarray hybridization values (log scale). ‘absent’ values were set to zero in this heatmap. Samples are HFF1 (-a and -b denote duplicates), 24, 48, 72 h, two HFF1-derived iPS cells (iPS2, iPS4) and two human ES cell lines (H1, H9). Illumina Ref-8 V3 microarrays detect the exogenous and endogenous forms of *OCT4* (*POU5F1*), since the microarray probe was located within the coding region of the *OCT4* transcript. Illumina probes for *SOX2, KLF4* and *c-MYC* were designed to the 3′UTR end, and therefore do not detect the virally expressed transcripts of these genes.

In order to gain molecular insights into the processes operative during the early stages of reprogramming, we profiled genome-wide transcriptional changes in HFF1 cells at 24, 48, and 72 h post-transduction of OSKM encoding viruses. The transcriptomes of these cells were compared to two HFF1-derived iPS cell lines (iPS2, iPS4) and the ES cell lines (H1, H9) as references of pluripotency. We detected exogenous protein expression of the OSKM factors as early as 24 h with successive increases at 48 and 72 h (Figure 1A). Of the reprogramming factors, endogenous forms of *KLF4*, and *c-MYC* could be detected on the microarrays (Figure 1B) and distinguished from exogenous transcripts, based on transcribed 3′UTR regions. Expression of endogenous *OCT4*/*POU5F1* could not be differentiated from its exogenous counterpart, as the Illumina probe is located exclusively within the coding region of this gene. Endogenously expressed *SOX2* was not detected at these time points.

### The transcriptomes of viral transduced cells become less fibroblast-like and more pluripotent-like over time

**Figure 2:**
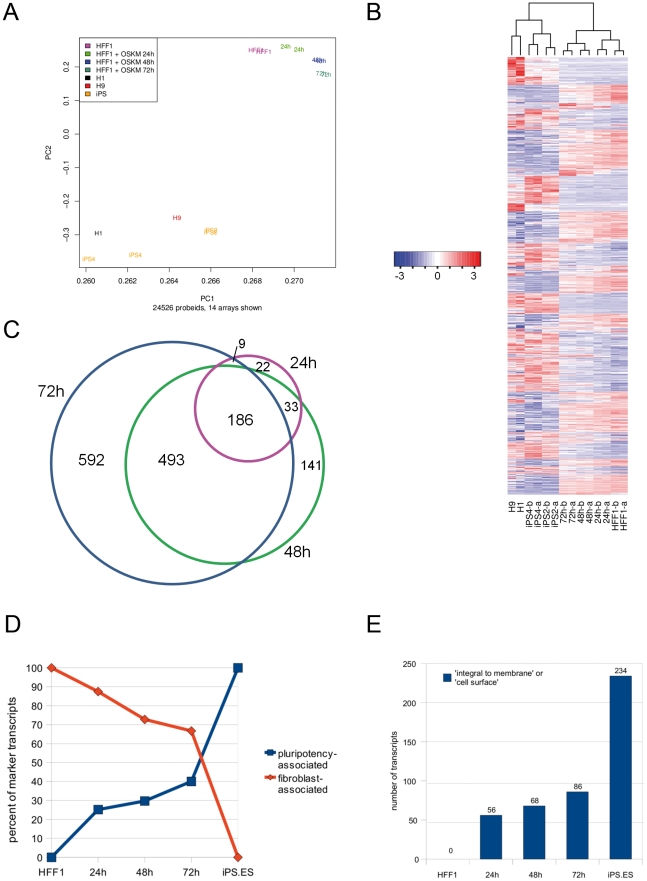
Contrasting transcriptomes of somatic, OSKM transduced HFF1 and pluripotent cell types. Expression profiles were obtained from donor cells (HFF1; human foreskin fibroblasts), donor cells transduced with OSKM at 24 h, 48 h and 72 h, HFF1-derived iPS cell lines (iPS2, iPS4) and ES cell lines (H1, H9). A. Principal component analysis shows the projection of the vectors of hybridization values (24526 probes) on the first two principal components. B. Differential regulation between HFF1 cells and each of the three timepoints (24, 48, and 72 h), iPS and ES cells was determined using the Bioconductor package limma (see Methods). The normalized expression values (z-score) of 6179 transcripts (Data S1) regulated at any timepoint or in iPS/ES cells with respect to HFF1 cells (p_adj_<0.05; fold change >1.5) are shown. C. Venn diagram depicting the overlap between regulated transcripts (1476) at each timepoint. D. Alterations in the number of pluripotency- and fibroblast-associated transcripts during the time-course towards an increasing pluripotent and decreasing somatic (HFF1) transcriptome. E. Increasing numbers of pluripotency-associated transcripts linked to the GO terms ‘integral to membrane’ or ‘cell surface’ are detected in the time series.

The microarray expression profiles distinctly separate the donor HFF1 cells and the OSKM-transduced HFF1 cells from the ES and iPS cell lines (Figure 2A, PCA plot), demonstrating that the OSKM-transduced cells in these early time points still retain a high level of transcriptional similarity to their donor cell-type. The expression profiles of the duplicate samples clustered similarly, exemplifying low variability between replicates (Figures 2A and 2B). The arrangement of the time series samples in these plots indicates changes in gene expression leading to the transcriptomes of these OSKM-transduced cells gradually diverging away from the parental HFF1 cells. Accordingly, based on regulated transcripts (p_adj_<0.05) with a fold change greater than 1.5, the number of regulated transcripts (with respect to HFF1) increased with time, from 250 transcripts at 24 h, 853 at 48 h and culminating at 1280 transcripts at 72 h (Figure 2C). The majority of the gene expression changes of earlier time points are maintained at the successive time points, as indicated by the inclusion of the sets in the Venn diagram.

To assess the extent to which the transcriptomes of OSKM-transduced HFF1 cells had transformed to that of a pluripotent cell, we defined a set of pluripotency-associated genes (794 transcripts; e.g. *DNMT3B, GDF3, LEFTY2, PDK1* and *PROM1*) as those that are "present" (microarray p_detection_<0.01) in both HFF1-derived iPS and ES cells but "absent" (p_detection_ ≥0.01) in non-transduced HFF1 cells. Similarly, we defined a set of fibroblast-associated genes that are "present" in non-transduced HFF1 cells but "absent" in both HFF1-derived iPS and ES cells (510 transcripts; e.g. *CD59, CD68* and *CD109*). The proportion of pluripotency-associated genes that are expressed at each time point increases with time, whilst the proportion of fibroblast-associated genes decreases (Figure 2D; Data S2). Moreover, Gene Ontology enrichment in cellular component terms showed that 29% of the pluripotency-associated genes were ‘integral to membrane’ or ‘cell surface’ proteins (e.g. *CD83*, *CD24*, *PDPN*), which were increasingly ‘switched on’ over time (Figure 2E; Data S2). Five of these genes (*HAS3*, *SLCO4A1*, *PODXL*, *PDPN*, and *F11R*) encode proteins that have been identified as cell surface markers of undifferentiated human ES cells [23] and therefore could serve as antigens for fluorescence activated cell sorting (FACS) enrichment in order to pre-select OSKM-transduced cells that already express human ES cell markers, prior to plating onto feeder cells and further culturing under conditions that support the undifferentiated propagation of human ES cells.

### Gene Ontology enrichment of regulated transcripts identifies functions operative in early reprogramming

**Figure 3:**
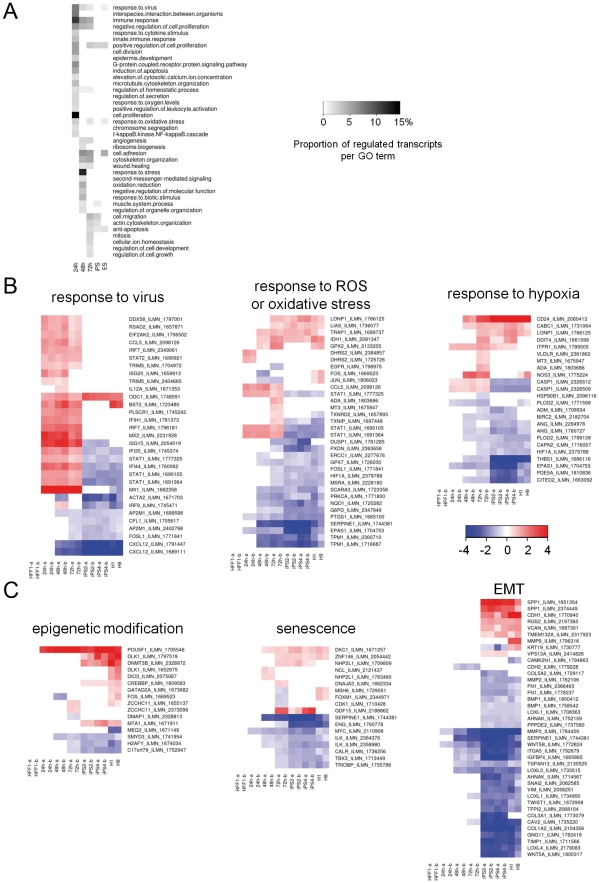
GO categories enriched in regulated transcripts. A. For selected GO categories, the fraction of transcripts in each GO category compared to the number of regulated transcripts is depicted as a heatmap. Colours indicate no enrichment (white) or proportion of enriched transcripts (shades of black to white), as a percentage of regulated transcripts at each timepoint. B. Regulated transcripts (fold change >1.5; p_adj_<0.05) in selected GO categories are shown. Colours indicate the log2 fold change, either up-regulated (red), down-regulated (blue), or not regulated (white) with respect to HFF1 cells. Heatmaps shown are transcripts from stress-related GO categories (panel B) or groups of genes known to have an influence on reprogramming (panel C). Other categories are shown in Figure S1.

We looked for enrichment in Gene Ontology (GO) biological process terms of regulated transcripts (p_adj_<0.05) between HFF1 cells and the three time points (see Data S3). “Response to virus” and “immune response” GO categories were prominently over-represented in all time points (Figure 3A), consistent with the stress induced by viral infection. Many of the transcripts regulated in these categories are acutely and specifically induced within the first 72 h of transduction but not in the HFF1-derived iPS (Figure 3B) suggesting that this is a transient effect. Following the initial immune response, there are strong regulations in GO groups related to response to physiological oxygen (“response to reactive oxygen species”, “oxidative stress” and “response to hypoxia”; Figure 3B), apoptosis, cell proliferation, cell cycle, cellular morphological changes and aging (Figure S1).

We then examined whether particular pathways were activated or repressed during the time series. To achieve this we clustered the genes by their expression pattern (fuzzy c-means; Figure S2) and then examined the clusters for KEGG pathway enrichment and perturbation using signalling pathway impact analysis (SPIA; [24]; Data S4). The most significant result was obtained for a cluster of 195 genes that describes transcripts highly up-regulated in the time series but down-regulated in HFF1-derived iPS and ES cells. This cluster suggests three perturbed pathways: the pathway “Focal adhesion” (p_GFDR_<0.05 at 48 and 72 h; Figure S3), reflecting the nature of adherent cell culture and the potential establishment of cell-cell contact conducive for inducing pluripotency; the “TGF-beta signaling pathway” (p_GFDR_<0.1 at 72 h), which is active in epithelial-to-mesenchymal transition; and the “Malaria” pathway (p_GFDR_<0.1 at 24 h; p_GFDR_<0.05 at 48 and 72 h), which encompasses genes involved in innate immune response such as *STAT1, STAT2* and *MX2* (see Figure S5).

### Specific functional groups pertinent to reprogramming show minimal changes during the first three days of reprogramming

In the subsequent paragraphs we describe changes in expression of genes related to the process of reprogramming. We observed that the number of pluripotency-associated genes expressed is moderate. However, we noted genes that are already differentially expressed and could be targets for optimizing the reprogramming protocol. Here we focus on genes with functions related to epigenetic modification, senescence and epithelial-to-mesenchymal transition (Figure 3C).

### Epigenetic modification

Previous studies related to the epigenetic control of reprogrammed cells, either by somatic cell nuclear transfer (SCNT) or by factor-induced pluripotency, revealed that global inhibition of DNA methylation activity and inhibition of histone deacetylation increase reprogramming efficiency [25], [26], [27], [28], [29], [30]. In particular, treatment of OSKM-transduced mouse embryonic fibroblasts (MEF) with the DNA methylase inhibitor 5′azaC for one week post-infection, increased reprogramming efficiency [31]. Therefore, gene regulation leading to DNA de-methylation during the early stages of reprogramming favours the induction of pluripotency. Consistent with this, we observed that the SET and MYND domain-containing protein 3 (SMYD3), a histone methyltransferase that can specifically methylate histone H3 at lysine 4 and activate the transcription of a set of downstream genes [32] is down-regulated at 48, 72 h and also in iPS and ES. Likewise, C17orf79 (which modulates the histone H4 methylation activity of PRMT5 [33]) and histone H2AFY are also down-regulated at 72 h and also in iPS and ES.

The pluripotency factor LIN28 and let-7 miRNA negatively regulate each other. ZCCHC11 is a terminal uridylase that is recruited to let-7 miRNA by LIN28, leading to uridylation of pre-let-7 and its inactivation [34], [35], thereby de-repressing LIN28. We see an up-regulation of ZCCHC11 at 72 h, which could support expression of LIN28.

Methylation of the imprinted Dlk1-Dio3 locus (*DLK1*, *MEG3*, *DIO3*) has been associated with impaired pluripotency in mouse iPS cells [36]. We observed that *DLK1* is a pluripotency-associated gene (Data S2) that is already switched on at 72 h (present; microarray p_detection_<0.01), although not detected as differentially expressed at 72 h according to our cut-offs. Both *DLK1* and *DIO3* are up-regulated in ES cells, although *MEG3* is down-regulated (Figure 3C).

### Senescence

The activation of senescence presents a roadblock during the reprogramming process [37], therefore we investigated whether genes related to senescence were differentially expressed during the time series. Only a minor fraction of senescence related genes (18 from a total of 117 as defined in [21]) were differentially expressed during the time series (Figure 3C), suggesting that senescence is not favoured within the early reprogramming stages. Genes known to trigger senescence such as mTOR [38] or pro-inflammatory cytokine TNF-alpha [39] were not differentially expressed within the time series.

Most of these genes adopt gene expression changes coherent to their expression in the pluripotent samples and therefore their differential expression is likely to be positive for reprogramming. For example, we see the up-regulation within the time series and in the pluripotent cell lines of *DKC1* and *ZNF146*, which are involved in telomere maintenance [40], [41], and down-regulation of *SERPINE1*, a p53 target gene that is up-regulated in senescent compared to non-senescent cells [42]. Overall, the senescence expression profile of the time series and iPS cells is mostly coherent with that of the ES cells.

Interestingly, we observe a few senescence-related genes that are up-regulated in the time series but not in the ES cells (Figure 3C). Such genes may represent problematic spurious remnants of the reprogramming procedure and could be triggering senescence events happening at later stages in the reprogramming. The most conspicuous one is *GDF15*, which is also highly up-regulated in the virally transduced iPS and already at 72 h, but not in ES cells. GDF15 encodes a member of the transforming growth factor-beta (TGF-beta) superfamily, has pro-apoptotic activity and is induced by p53 [43].

### Epithelial-to-Mesenchymal Transition

Reversal of EMT, i.e., mesenchyme-to-epithelial transition (MET), also plays a major role in reprogramming somatic cells. ES cells are epithelial in nature whereas fibroblasts are of mesenchyme origin. In the process of reprogramming fibroblasts to iPS cells, fibroblasts must be converted into a more epithelial-phenotype via MET. MET can be promoted by suppressing the opposite process, EMT. The process of EMT is essential for gastrulation to occur and is driven by TGFB1, which ultimately inhibits the expression of E-Cadherin, through SMAD signaling. Initial inklings implicating MET in fibroblast reprogramming were demonstrated by Lin and co-workers [11], who showed that TGF-beta inhibited OSKM reprogramming of human fibroblasts, while use of a TGF-beta receptor inhibitor (SB431542) increased reprogramming efficiency. Further studies have shown that the endpoint of MET, E-Cadherin (CDH1), is required for establishing cell-cell contacts critical for the iPS phenotype [44]. Finally, two independent groups definitively showed that MET was required for initiating and maintaining the reprogramming of MEF cells, and that OSKM factors played a role in this transition by suppressing Snail (Sox2/Oct4) or TGF-beta receptors (c-Myc) or up-regulating epithelial genes, including E-Cadherin (*CDH1*) [45], [46]. Additionally, BMP signaling was shown to contribute to reprogramming of MEF cells by enhancing expression of miRNAs that either promote expression of epithelial-associated genes (*Cdh1*, *Epcam, Ocln*) or repress inhibitors of EMT (*Zeb1/Zeb2, Snail, Slug*) [46].

The results from the time series show that although some EMT-related genes are down-regulated in the time series (Figure 3C), the endpoint of MET activation, as indicated by the up-regulation of *CDH1* (evident in iPS and ES cells; Figure 3C), had not yet occurred within the first three days of OSKM-mediated fibroblast reprogramming. Given that reprogrammed mouse fibroblasts first show *CDH1* expression after six days of OSKM induction and that the total reprogramming time is ∼20 days in mouse [45] and ∼30 days in human, it is reasonable that we do not yet see *CDH1* up-regulation after three days in human fibroblasts. However, within the first three days, we do observe changes in *CDH2* (N-Cadherin), which is activated during EMT and proposed to be a functional switch between focal adhesion and cell-cell adhesion during EMT [47]. *CDH2* is down-regulated in our dataset (at 24, 48, 72 h compared to HFF1; Figure 3C), which could indicate the start of switching to a cell-cell adhesion morphology.

The lysyl oxidase family (LOX, LOXL1-4) oxidizes the side chain of lysine to its aldehyde, releasing NH_4_^+^ and H_2_O_2_ [48]. Originally shown to be involved in stabilizing the extracellular matrix by catalysing covalent links between collagen and elastin, other functions for lysyl oxidases have since been discovered. During EMT, LOXL2 and LOXL3 synergise with Snail to repress E-cadherin expression [49]. In this regard, we observe the down-regulation of *LOXL3* at 48 h, 72 h, and in iPS and ES cells, supporting a move towards de-repression of E-Cadherin in favour of MET.

### Signature of EMT suppression

In summary, many pluripotency-associated genes are not yet active between 24 to 72 h post transduction but we find traces of expression changes related to pluripotency. We wondered if we could detect a larger signature involving genes triggering reprogramming by suppression of EMT, which might be transiently up-regulated during reprogramming and silent in pluripotent cells. Such a signature is supported by the comparison of the genome-wide EMT-ranked list with the down-regulated genes at each time point. Rank correlation analysis (see Methods for details; [50]) revealed that EMT is increasingly suppressed during reprogramming towards iPS and in ES cells (Table 1). A very similar result was obtained for an alternative approach using the binomial test to assess the over-representation of EMT-related genes in the down-regulated genes during reprogramming (Table 1; Figure S4).

**Table 1:** Rank correlation and binomial test shows suppression of positive EMT-related genes during reprogramming.

	Spearman rank correlation		Binomial test
Cell type	rho	p-value	p-value
24 h	0.187	0.54	0.582
48 h	0.197	0.049	1.17e-05
72 h	0.219	0.005	3.98e-08
iPS	0.349	2.78e-14	7.82e-05
ES	0.367	3.71e-15	0.020

### Integration of OSKM interaction networks reveals potential avenues for improving the reprogramming protocol

**Figure 4:**
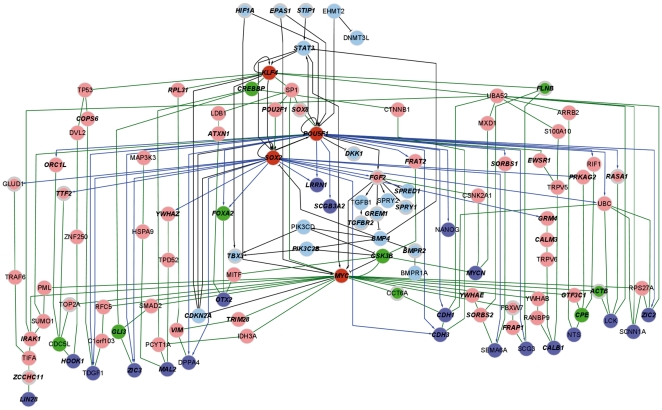
Integrated network of the reprogramming process. An integrated network was constructed by joining subnetworks of the OSKM regulators and downstream interaction networks. The OSKM upstream regulator subnetwork (light blue nodes connected by black lines) consisted of OSKM (red coloured nodes) and included regulators of OSKM (see Methods for details). The downstream interaction network was assembled using the reprogramming factors as sources (OSKM; red coloured nodes) and a list of regulated genes between fibroblasts and iPS or ES cells, obtained from a meta-analysis of five iPS studies as sinks (purple coloured nodes; [17]). Connections between sources and sinks were determined by protein-protein interactions and regulatory interactions from the POU5F1 and SOX2. Nodes were scored for their centrality and labeled accordingly, green: high-scoring nodes; pink, low scoring nodes (see Methods for details). Blue and green edges represent regulatory interactions and protein-protein interactions, respectively. Genes that are regulated at one or more time points (24 h, 48 h, 72 h) are circled in gray and labels for genes that are regulated in iPS or ES cells are in boldface italics.

We hypothesize the existence of pluripotency amplification pathways whose activation would be required for successful reprogramming. To identify key components of these pathways, we generated an interaction network that connected the OSKM factors to genes specifically expressed in iPS and human ES cells and included upstream regulators of OSKM (Figure 4; see Methods for details).

As a highly connected hub in the interaction subnetwork, GSK3B was closely examined for relevance to reprogramming. GSK3B is involved as an intermediate in four different paths from the sources to sinks, starting from c-MYC or POU5F1 and ending at MYCN or OTX2. GSK3B itself is a kinase, whose activity is dependent on phosphorylation on Ser-21. Its substrates include c-MYC, MYCN and CTNNB1; their phosphorylated forms lead to their degradation [51], [52]. We focus on the following path: POU5F1-FRAT2-GSK3B-MYCN. In the first step, viral transduction of POU5F1 positively regulates FRAT2 expression by binding to its promoter [53]. Next, FRAT2 promotes Wnt signaling by competing with GSK3B for binding to AXIN, thereby interfering with the destruction complex required to phosphorylate CTNNB1 and mark it for proteomic degradation [54]. In the next step, GSK3 inhibition exhibits multiple roles. Inhibiting GSK3 activity appears to antagonize differentiation in ES cells and promote reprogramming of somatic cells. The use of a GSK3 inhibitor, 6-bromo-indirubin-3′-oxime (BIO), under feeder-free conditions, supports the maintenance of human ES cells in an undifferentiated state [55], [56]. Additionally, human hair follicle outer root sheath cells treated with BIO appear to be more undifferentiated in comparison to untreated cells [57]. It is thought that GSK3 inhibition maintains human ES cells in an undifferentiated state by preventing EMT [58], which is essential for gastrulation and subsequent formation of the three germ layers. Moreover, in the context of reprogramming by somatic cell fusion with embryonic cells (mouse), activation of the WNT pathway in fused hybrid cells by either GSK3 inhibitor or WNT3A ligand increased reprogramming efficiency [59]. The final target of WNT signaling, CTNNB1, is stabilized upon GSK3B inhibition, leading to a cytoplasmic accumulation of CTNNB1 and translocation to the nucleus, where it initiates transcription of target genes. In the context of reprogramming, there are presumably CTNNB1 targets that drive reprogramming [59]. Finally, in the last step, MYCN is a target for protein degradation by GSK3-dependent phosphorylation [52], however, in the absence of GSK3 kinase activity, MYCN accumulates and is able to activate transcription of DNMT3A [60].

In support of this path, we observed up-regulation of *FRAT2* in iPS and ES cells (log2FC  = 1.5 and 2.3, respectively) and the down-regulation of *GSK3B* transcript at 48 h, 72 h and in iPS and ES cells (log2FC  = −0.54, −0.55, −1.1, and −0.45, respectively). Although we cannot ascertain the concentration of active GSK3B kinase present at these time points from microarray data, the observed decrease in transcript limits *de novo* protein synthesis of GSK3B protein and possibly impacts GSK3B function by restricting protein availability. We also observe a strong up-regulation of MYCN in iPS and ES cells compared to HFF1 cells (log2FC  = 1.6 and 2.7, respectively) and a weak up-regulation of DNMT3A in ES cells (log2FC  = 0.36).

In summary, POU5F1 targets *FRAT2* transcription. FRAT2, in turn, promotes WNT signaling. Additionally, diminished kinase activity of GSK3B promotes reprogramming by: 1) antagonizing differentiation and promoting the undifferentiated state by inhibiting EMT; 2) activating WNT pathway, leading to transcription of yet unknown CTNNB1 target genes that promote reprogramming; 3) stabilizing MYCN protein levels, which drives expression of DNMT3A, a *de novo* methyltransferase that is highly expressed in iPS and ES cells. Together, this path supports epigenomic changes mediated through WNT signaling.

We also note a path in our network through two highly connected genes, including an activator of NANOG, another pluripotency factor, therefore constituting yet another pluripotency amplification path: KLF4-CREBBP-GLI3-ZIC3 (Figure 4). KLF4 can be acetylated by CREBBP, which then enhances transcription of KLF4 target genes [61]. CREBBP, GLI3 and ZIC3 are all transcription co-activators/repressors. In particular, ZIC3 is known to be specifically and highly expressed in undifferentiated ES cells, and represses endodermal differentiation by activating NANOG [62]. Based on binary interactions between CREBBP-GLI3 [63], [64] and GLI3-ZIC3 [65], [66], it seems plausible that they could act co-ordinately to promote pluripotency. In support of this, we observed the up-regulated expression of *CREBBP, GLI3*, and *ZIC3* in iPS and ES cells.

### Viral transduction of HFF1 cells initiates a cascade of events that ultimately leads to the activation of p53

Irrespective of the reprogramming favouring events we observed at 72 h, such as the activation of some pluripotency amplification paths and partial suppression of EMT, the cells are far from a pluripotent state. At this early phase of reprogramming, the most evident gene expression signature is related to the adverse effects of viral transduction (Figure 3). Since this response might affect the effectiveness of reprogramming, we hypothesize that characterization of this response might lead to improvements in the reprogramming protocol based on limiting the negative effects of the reprogramming protocol itself.

In relation to this, we observed the early and progressive activation of genes related to anti-viral responses, response to ROS and DNA damage. We propose that the viral response results in the generation of ROS, which ultimately triggers DNA damage and p53 activation, leading to an apoptotic response with the result of a reduced efficiency of the reprogramming process.

First, we confirmed by real time-PCR the significant up-regulation of genes involved in innate immunity in response to viral infection (e.g. *CCL5, IRF7, STAT2, TRIM5, DDX58, MX2, IL12A, EIF2AK2* and *ISG20*) and induction of apoptosis (e.g. *IL19, NGEF, STAT1* and *CASP1*) in cells transduced with virus in comparison to untreated fibroblasts (Figure S5).

Next, we tested the effect of viral transduction on ROS production. HFF1 cells were transduced with the Yamanaka factors (OSKM) or a vector expressing GFP. As a negative control, cells were treated with polybrene, an additive that is used as part of the viral transduction protocol.

**Figure 5:**
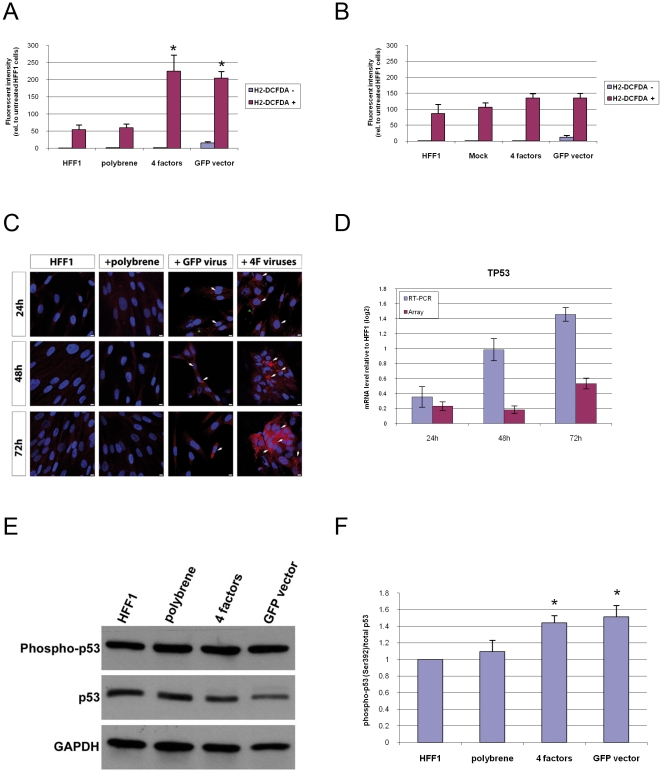
Cellular responses to retroviral transduction. A. Quantitative analysis of reactive oxygen species (ROS) production in HFF1 cells, polybrene-treated HFF1 cells, OSKM (4 factor)-transduced HFF1 cells, and GFP-transduced HFF1 cells under retroviral transduction conditions. We measured ROS production 24 h after transduction by flow cytometry using 2′,7′-dichlorofluorescin diacetate (H2-DCFDA) in two independent experiments. Values are presented as relative changes compared to HFF1 cells without H2-DCFDA treatment. ROS levels from 4 factor-transduced HFF1 cells and GFP-transduced HFF1 cells are significantly up-regulated compared to HFF1 cells (significant changes: *, p<0.05). B. Quantification of ROS levels in HFF1 cells, mock cells, 4 factor-transduced HFF1 cells, and GFP-transduced HFF1 cells under nucleofection conditions. Measurements are as described in 5A. C. DNA damage in HFF1 fibroblasts. HFF1 cells were either left untreated or exposed to polybrene only, or to GFP- or OSKM-encoding virus. DNA damage was assayed by 8OHdG immuno-staining and monitored at three different time points (24 h, 48 h, and 72 h). Untreated fibroblasts or fibroblasts exposed to polybrene only, did not show any accumulation of DNA damage. In contrast, HFF1 transduced with GFP or 4 factors exhibited foci of nuclear and mitochondrial DNA damage (white arrows). HFF1 transduced with OSKM tended to cluster in cellular aggregates over time and showed a higher level of DNA damage. At 24 h, we observed the presence of small DAPI-positive dots in all transduced fibroblasts, which may correspond to viral DNA (green arrowheads). Magnification used was 63X, scale bar corresponds to 10 µm. D. Level of *TP53* expression at 24, 48, and 72 h post-expression transduction of OSKM as measured by hybridization of the array of in real-time PCR confirmation. E. Western blot showing expression levels of phosphorylated p53 and non-phosphorylated p53 in untreated HFF1 cells, or HFF1 cells treated with polybrene, transduced with viruses expressing OSKM or GFP at 24 h post-transduction in two independent experiments. F. The ratio of expression values of phospho-p53 versus total p53 is presented as relative changes compared to untreated HFF1 cells for polybrene-treated cells, 4 factor-transduced HFF1 cells, and GFP-transduced HFF1 cells (significant changes: *, p<0.05).

We then analyzed ROS production at 24 h post-transduction. ROS levels in polybrene-treated HFF1 cells were similar to that in untreated HFF1 cells. On the contrary, retroviral transduction resulted in significantly increased levels of ROS. In addition, we did not observe a significant difference in ROS levels between OSKM-transduced HFF1 cells and GFP-transduced HFF1 cells (Figure 5A). To test the possibility that the exogenous DNA could also trigger ROS production, we simultaneously performed nucleofection-based transfections using the same vectors. Nucleofection reactions without vector DNA (mock control), with four vectors each expressing OSKM, and a vector expressing GFP did not significantly affect the production of ROS as compared to HFF1 cells (Figure 5B). However, we must point out that the efficiency of nucleofection was slightly lower than viral transduction (Figure S6), so it cannot be completely excluded that the nucleofection reactions did not modulate the levels of ROS.

We further tested the effect of viral transduction on DNA damage. Nuclear and mitochondrial DNA damage was monitored over time using 8OHdG immuno-staining (Figure 5C). Untreated HFF1 cells or HFF1 cells exposed to polybrene only, did not show accumulation of DNA damage. On the other hand, HFF1 transduced with viruses expressing GFP or OSKM (4F) exhibited foci of nuclear and mitochondrial DNA damage at 24 h post-transduction. At this time point, we also observed the presence of small DAPI-positive dots in all transduced fibroblasts, which may correspond to viral DNA. Over time, the foci of DNA damage appeared to increase in both GFP and OSKM-transduced HFF1 cells. Overall, HFF1 transduced with OSKM, which showed a tendency to cluster in cellular aggregates over time, exhibited the highest level of DNA damage.

As our experiments demonstrated viral transduction-induced DNA damage, we next investigated if this leads to the activation of p53. At the mRNA level, real-time PCR confirmed an increasing expression of *TP53* transcript from 24 to 72 h (Figure 5D). We additionally analyzed protein expression levels of phosphorylated p53 and non-phosphorylated p53 within the same cells used for measuring ROS levels. As shown in Figure 5E and 5F, untreated and polybrene-treated HFF1 cells had similar levels of phosphorylation of p53. In contrast, phosphorylation of p53 was more pronounced in OSKM- and GFP-transduced HFF1 cells than in untreated HFF1 cells. We did not observe significant differences in the levels of phosphorylated p53 between OSKM- and GFP-transduced HFF1 cells.

**Figure 6:**
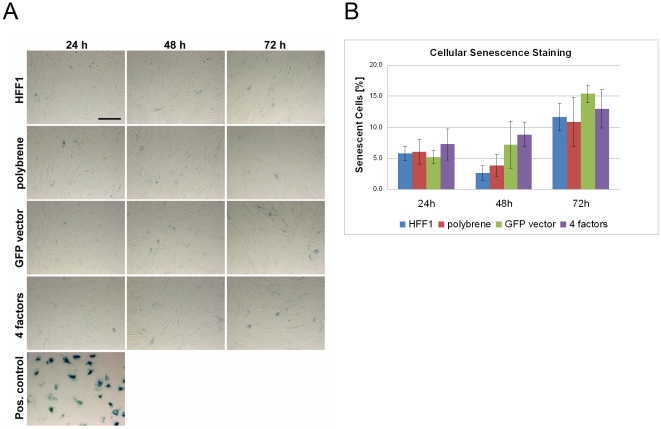
Detection of cellular senescence. A. Bright-field images of HFF1 cells after staining for senescence beta-galactosidase activity (scale bar: 200 µm). B. Summary of the quantification of blue-stained senescent cells as a percentage of the total number of cells analyzed.

Following up on our results demonstrating the production of ROS and activation of p53 upon viral transduction, we asked if senescence had also been activated at the cellular level. We previously observed the early regulation of some senescence-related genes at the transcriptional level (Figure 3C), which parallel the changes seen in pluripotent cells. Indeed, the cellular senescence assays confirm the transcription results from Figure 3C. Although senescence was detectable in the analyzed HFF1 samples, as indicated by weak blue β-gal staining, the intensity of the staining and the proportion of stained cells were much lower compared to senescence-prone amniotic fluid cells, which served as the positive control (Figure 6A). The percentage of senescent cells in the viral transduced HFF1 samples (GFP, 4 factors/OSKM) gradually increased between 24 h to 72 h post-transduction (Figure 6B). However, these differences were not significant when compared to untreated HFF1 cells. Owing to the weak overall staining intensities, which should most probably be considered as background noise, the observed differences appear negligible. Potentially, it was too early in the reprogramming process to detect senescence.

We reasoned that suppressing innate immune response to viral transduction could have a positive impact on reprogramming by modulating ROS production, DNA damage and subsequent p53 activation. B18R is a recombinant protein derived from vaccinia virus that binds Type I interferons and has been shown to neutralize anti-viral responses in cells [67]. To this end, we investigated the effect of B18R supplementation on reprogramming efficiency.

**Figure 7:**
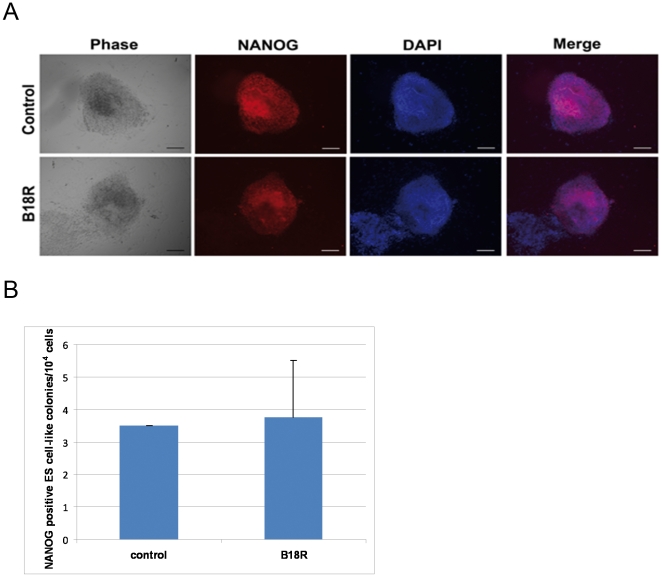
Effect of B18R protein on reprogramming. A. NANOG-positive iPS colonies as shown by immunofluorescence staining with and without B18R. Scale bars represent 100 µm. B. Comparison of the total number of NANOG-positive iPS colonies obtained with and without B18R supplementation.

We observed that the concentration (200 ng/ml) of B18R used in our experiment yielded NANOG-positive iPS cells; i.e. supplementation of this reagent was not toxic to the cells. However, adding B18R did not result in an increase in the number of NANOG-positive iPS colonies as compared to non-supplemented OSKM-transduced HFF1 cells (Figure 7). There might be innumerable reasons for the lack of success of this application. We believe that testing other modulators of innate immune responses would be a promising avenue for improving the efficiency of inducing pluripotency in somatic cells.

## Discussion

Viral transduction of reprogramming factors remains the most robust method to introduce immediate and high level expression of exogenous OSKM factors for deriving iPS cells from somatic cells. Despite this, we do not fully understand the mechanisms underlying the molecular, immunological and biochemical pathways leading to the induction of pluripotency. To this end, we have used microarray-based transcriptome analysis to identify crucial events occurring within the first 72 h of initiating reprogramming. On the one hand, we find that processes known to play a role in reprogramming appear to be partially initiated (MET, epigenetic modification), some ES cell surface antigens are expressed, and a pathway involving pluripotency-associated factors and *GSK3B* is activated. On the other hand, we found that the dominating effect observed at the time points analyzed is activation of innate immunity in response to viral transduction.

Somatic cell reprogramming by viral transduction is a double-edged sword. Despite the protocol's robustness, the host cell viral response acts as a roadblock to efficient reprogramming by initiating a damaging and repressive chain of events, namely ROS production, DNA damage, activation of p53 and senescence.

Following our finding from the gene expression analysis that the viral response is highly up-regulated within the first three days of the reprogramming protocol, we then demonstrated that the mere process of viral transduction elicits the expression of genes involved in innate immunity, whereas this effect is minimal upon the transfection of plasmids into HFF1 cells. Furthermore, HFF1 cells subjected to viral transduction, as opposed to nucleofection of plasmids encoding OSKM, exhibited increased concentrations of ROS. DNA damage also ensued in viral-transduced cells compared to control cells. We also observed that anti-oxidant genes were not up-regulated during viral transduction, which may indicate that the cells are vulnerable to ROS-induced damage.

Curbing elevated ROS levels could be beneficial for reprogramming because ROS has been shown to promote differentiation of ES cells [68]. Moreover, the use of ROS scavengers, along with hypoxic growth conditions has been shown to promote de-differentiation in human adipose stromal cells [69]. Additionally, a metabolomic study of ES cells suggests that intracellular redox state and hypoxia regulate differentiation and self-renewal [70]. Furthermore, the anti-oxidant vitamin C has been shown to enhance the efficiency of inducing pluripotency in somatic cells [71]. Therefore, buffering rapid increases in ROS by using an anti-oxidant may be beneficial in the early stages of reprogramming by counteracting differentiation and preventing ROS-induced damage, thereby increasing the efficiency of reprogramming.

It has been demonstrated that viral infection elicits DNA damage in host cells [72]. This might be deleterious to reprogramming. Previous studies have shown that the efficiency of iPS derivation can be improved by inhibiting p53, which inactivates the host cell's natural repair response to DNA damage. However, iPS cells obtained in this manner are susceptible to chromosomal aberrations [73]. We suggest that upstream intervention to avoid p53 activation in the first place can be beneficial to the reprogramming progress. Conceivably, dampening the initial and rapid host cell response to viral infection could be an effective means to achieve this.

**Figure 8:**
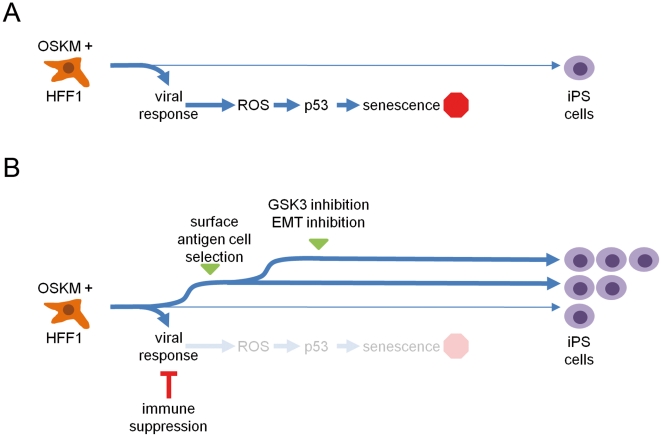
Model for improving the OSKM-based reprogramming protocol. A. Standard protocol results in relatively few iPS cells. B. Suppression of the initial viral response, combined with FACS-based enrichment of cells expressing ES-specific cell surface receptors and inhibition of GSK3 and EMT, could increase reprogramming efficiency.

The role of immune response is becoming an important consideration during the reprogramming process. A previous study has shown that anti-inflammatory molecules promote self-renewal in ES cells [74], which may also apply to iPS cells. Furthermore, recent developments in RNA transfection protocols have used immune suppression to increase RNA transfection efficiency [15]. Interferon inhibition, combined with synthetic RNA that has been modified to evade host defense mechanisms against ssRNA, yield iPS cells in an efficient manner [18]. Together, these studies suggest that attenuation of the donor cell's immune response is beneficial to the reprogramming process. However, supplementing OSKM encoding viruses with 200 ng/ml of the interferon inhibitor B18R did not increase the efficiency of reprogramming but also did not have an adverse effect on the induction of pluripotency in HFF1 cells. Despite this, we believe that transient suppression of innate immunity could be a step towards modulating ROS and ultimately p53 levels, resulting in increased reprogramming efficiency. This coupled to activation of pluripotency amplification pathways and EMT suppressors and pre-selecting for cells expressing ES cell surface antigens such as PODXL are complementary strategies (Figure 8) for increasing the efficiency of deriving iPS cells as suggested by this study.

## Materials and Methods

### Cell culture and viral transduction

Human neonatal foreskin fibroblast-HFF1 cells (ATCC) and Phoenix™ Ampho cells (Orbigen, Inc.) were maintained in Dulbecco's modified Eagle medium (DMEM, Gibco) containing 10% fetal bovine serum (Invitrogen) and 0.5% penicillin and streptomycin (Invitrogen). Human ES and iPS cells were maintained on irradiated mouse embryonic fibroblasts (MEF) cells in KnockOut DMEM (Invitrogen) supplemented with 20% KnockOut serum replacement (Invitrogen), 0.1 mM non-essential amino acids (Invitrogen), 1 mM L-glutamine (Invitrogen), 0.1 mM β-mercaptoethanol (Sigma), 0.5% penicillin and streptomycin and 4 ng/ml basic fibroblast growth factor (bFGF, Invitrogen) as described by [21]. The human ESC line was purchased from WiCell Research Institute (Madison, WI, USA, www.wicell.org).

pMX-based retroviral vectors each encoding the transcription factors OCT4, SOX2, KLF4, and c-MYC were transfected into Phoenix™ Ampho cells using the Fugene transfection reagent (Roche). Viral supernatants were collected 48 and 72 h post-transduction. HFF1 cells were incubated with virus supernatants supplemented with 4 µg/ml polybrene (Sigma) in duplicate and then centrifuged at 800 rcf for 99 min. The transduced cells were harvested 24, 48, and 72 h post-transduction.

To investigate the effect of type I interferon inhibition on the efficiency of reprogramming, we incubated HFF1 cells with virus supernatants supplemented with 200 ng/ml B18R protein (eBioscience, San Diego, CA, http://www.ebioscience.com, #14-8185). After 24 h, the cells were incubated with virus supernatants supplemented with B18R protein once more and then 24 h later, the infected cells were re-seeded onto irradiated MEF feeder layers (1×10^4^ cells/well, 12-well plate) in HFF1 cell culture medium supplemented with B18R protein. On the following day, the medium was changed to human ES cell culture medium, supplemented B18R protein. The medium was changed every other day. The chemical treatment was continued for 10 d. Following 10 days of treatment, the medium was replaced with MEF-conditioned medium supplemented with 4 ng/ml bFGF. On day 20 post-transduction, the cells were fixed and the number of NANOG positive ES cell-like colonies was counted.

### Immunofluorescence based staining

For immunofluorescence-based detection of expression of OSKM, cells were fixed in 4% paraformaldehyde (Science Services) in PBS for 20 min, permeabilized by 0.1% Triton X-100 (Sigma) for 10 min at room temperature and blocked with 10% chicken serum (Vector Laboratories) for 30 min. Thereafter, the cells were incubated with the specified primary antibodies overnight at 4°C. The primary antibodies included monoclonal antibodies against OCT4 (1∶100, Santa Cruz Biotechnology Inc., Santa Cruz, CA, http://www.scbt.com, #sc-8629), SOX2 (1∶100, Santa Cruz Biotechnology Inc., #sc-17320), KLF4 (1∶100, Santa Cruz Biotechnology Inc., #sc-20691), c-MYC (1∶100, Santa Cruz Biotechnology Inc., #sc-764), and NANOG (1∶500, Abcam, Cambridge, U.K., http://www.abcam.com, #ab62734). Secondary antibodies used were conjugated with either Alexa 488 or Alexa 594 (1∶300, Invitrogen, #A11055, A21442), which were incubated with the cells for 1 h at room temperature in the dark. Nuclei were counter-stained with DAPI (200 ng/ml, Invitrogen # H3570). Samples were analyzed on a Zeiss fluorescence microscope (Carl Zeiss, Oberkochen, Germany, www.zeiss.de).

For immunofluorescence-based detection of DNA damage, cells were fixed with 4% paraformaldehyde in PBS for 20 min at room temperature, washed twice with PBS and blocked with 10% chicken serum and 0.1% Triton X-100. Nuclei were counter-stained with DAPI. Primary antibody was 8-OHdG (1∶100, Millipore #AB5830) and the secondary antibody used was conjugated with Alexa 594 (1∶300, Invitrogen, #A21468). Coverslips were mounted using Dako fluorescent mounting medium (Dako #S3023) and visualized using a confocal microscope LSM 510 (Zeiss) at 63X magnification. The same parameters were applied to all samples. DAPI and 8OHdG images were always representative of the same single layer.

### Microarray-based transcriptome analysis

Total RNA was extracted using the MiniRNeasy Kit (Qiagen, Hilden, Germany), digested with DNase I (RNase-free DNase set, Qiagen, Hilden, Germany) following the manufacturer's instructions and quality checked by Nanodrop analysis (Nanodrop Technologies) and agarose gel electrophoresis. Approximately 500 ng of DNase I-treated RNA served as input for biotin-labeled cRNA production using a linear amplification kit (Ambion). Hybridizations, washing, Cy3-streptavidin staining, and scanning were performed on the Illumina BeadStation 500 platform (Illumina), according to manufacturer's instruction. cRNA samples were hybridized onto Illumina human-8 BeadChips version 3. The intensity values for each probe were derived by the Illumina software. The microarray data is available at Gene Expression Omnibus (http://www.ncbi.nlm.nih.gov/geo/) under the accession number GSE28688.

Raw data were further processed using tools available from Bioconductor (version 2.6) [75]. Background correction and normalization were carried out using the lumi package [76]. Illumina probes were then filtered by the detection p-value, considering probes with p-value <0.01 as ‘present’ and all others ‘absent’. Illumina probes which were ‘present’ in at least both duplicates of one sample were used for further analysis. Differential expression with respect to HFF1 samples was determined using the limma package [77] in Bioconductor. Gene annotations were based on human genome version 19 (illuminaHumanv3 package), Gene Ontology enrichment was carried out using the topGO package [78], and enrichment in KEGG pathways was performed using the SPIA package in Bioconductor [24].

We found the fuzzy-c-means (FCM) clustering method to be most suitable for our analysis compared to hard partitioning algorithms. Unlike commonly used approaches like k-means and hierarchical clustering, FCM is a soft partitioning method. FCM can assign genes to more than one cluster and represent the closeness of genes to specific clusters by membership values. It provides a very robust clustering as it reduces the effect of noise on the clustering process by a fuzzyfication parameter m which we set to 1.7. Clustering was performed by the mfuzz Bioconductor package [79].

To characterize the impact of reprogramming on key genes of EMT, we performed the following analysis. A list of key up-regulated genes involved in EMT was obtained from http://www.sabiosciences.com (*AHNAK, BMP1, CALD1, CAMK2N1, CDH2, COL1A2, COL3A1, COL5A2, FN1, FOXC2, GNG11, GSC, IGFBP4, ITGA5, ITGAV, MMP2, MMP3, MMP9, MSN, SERPINE1, SNAI1, SNAI2, SNAI3, SOX10, SPARC, STEAP1, TCF4, TIMP1, TMEFF1, TMEM132A, TWIST1, VCAN, VIM, VPS13A, WNT5A, WNT5B*). These genes were used as input for the web interface TargetFinder (www.targetfinder.org) [50], which uses a seed-based sorting algorithm [80] to rank all genes in the human genome according to their similarity to a given input set of genes. The sorted list of all genes showed a strong enrichment with regard to positive EMT genes as indicated by a recovery test (p<10e-20). The genes of that list were rank correlated (Spearman rank correlation) with the most significantly down-regulated genes at each time point (starting with the most down-regulated). Furthermore, a binomial test was performed to test for over-representation of the top positive EMT-related genes in the list of the most down-regulated genes at each time point.

### Construction of an OSKM interaction network

A large integrated network was constructed consisting of protein-protein interaction (PPI) data selected according to collective experimental evidence (Schaefer *et al*, submitted) and gene regulatory information for OCT4/POU5F1 and SOX2 from experiments of chromatin immunoprecipitation in human ES cells [53]. A set of source and sink proteins were specified. Source nodes were defined to be the four reprogramming factors (OCT4, SOX2, KLF4, c-MYC), and sink nodes were a set of 28 genes found to be differentially regulated between fibroblasts and iPS or ES cells, taken from a meta-analysis of five published studies [17]. A sub-network connecting the two protein sets was determined by merging all shortest paths between the source and the sink proteins. High-scoring nodes in this subnetwork were identified by applying a variant of the network betweenness centrality notion: for each protein the number of passing shortest paths connecting the source with the sink nodes was counted. This number was weighted by the inverse of the average shortest path length passing through the node favouring a large number of short shortest paths.

### OSKM upstream network reconstruction

Based on literature, gene regulatory networks were defined for OCT4, SOX2, KLF4 and c-MYC respectively, including upstream regulators of these transcription factors. Literature mining was executed via the Genomatix Pathway System (GePS) (www.genomatix.de/en/produkte/genomatix-software-suite.html). Networks were defined by analyzing the regulatory impact of the most frequently co-cited genes on abstract level, i.e., two genes are deemed interacting when co-cited within one abstract. These data are accessible as an interactive graphml-format under http://www.genomatix.de/OSKM/. Next, we selected the subnetwork of genes differentially expressed at any point of the time series. The resulting network was manually curated to remove false positives and contained one large component of 27 connected genes and 7 unconnected genes. Finally, we added back all genes that directly connected the unconnected genes (*BMP4*, *STAT3*, *EHMT2*, and *TGFB1*).

### Nucleofections

All nucleofections were performed by using the Nucleofector II (Lonza, Basel, Switzerland) and Nucleofector Kit R / program U-20. HFF1 cells were harvested by trypsinization and counted cell pellets consisting of 5×10^5^ cells were resuspended in 100 µl Nucleofector Solution (including supplement) plus DNA. 4-factor nucleofection: 1.5 µg of each plasmid pMXs-hOCT3/4, pMXs-hSOX2 pMXs-hKLF4 and pMXs-hc-MYC. GFP control nucleofection: 1.5 µg of a plasmid expressing GFP (Lonza) adjusted to 6 µg by empty vector pcDNA3.1 (to allow an estimation of the nucleofection efficiency for a single factor). For the mock control, nucleofection was carried out using Nucleofector Solution (including supplement) without plasmids. In order to ensure equal conditions in terms of dilution of the Nucleofector Solution, the different concentrations of the individual plasmid stocks were considered by adjusting all nucleofection reactions to the same volume with distilled water. The mixtures were transferred to a cuvette and immediately nucleofected. Immediately upon nucleofection, 500 µl of pre-warmed cell culture medium was added to the cuvette and the whole suspension then gently transferred into pre-warmed cell culture medium. Cells from nucleofections of the same kind were pooled together and seeded for incubation in duplicates into 12-well-plates (cells in 2.5 ml culture medium/well; for ROS measurement). Approximately 24 h later, dead cells were removed from the attached cells by washing once with PBS (Gibco/Invitrogen, USA) and replacing the cell culture medium.

### Measurement of reactive oxygen species

Intracellular ROS production was measured by flow cytometry using 2′,7′-dichlorofluorescin diacetate (H_2_-DCFDA, Sigma, St. Louis, http://www.sigmaaldrich.com, D6883). Cells were loaded with 15 µM H_2_-DCFDA in PBS for 30 min. After washing twice with PBS, cells were trypsinized by phenol-red-free-trypsin (Invitrogen, Carlsbad, CA, http://www.invitrogen.com, 15400-054). The cells were washed with PBS twice and analyzed on a FACS Aria (Becton Dickinson). The data was analyzed using FlowJo (www.flowjo.com, Tree Star Inc., Ashland, OR). At least 10,000 cells of each sample were analyzed.

### Western blotting

Total cell protein extracts were obtained using a modified RIPA buffer (50 mM Tris pH 7.4, 100 mM NaCl, 10 mM EDTA, 1 mM PMSF, 1% IGEPAL) supplemented with a complete protease inhibitor cocktail (Roche Diagnostics) prior to use. Protein concentration was determined according to the Bradford method. Proteins (20 µg) were resolved by electrophoresis on 7 % sodium dodecyl sulphate-polyacrylamide gel and transferred to nitrocellulose membrane (GE Healthcare Life Sciences, Piscataway, NJ, http://www.gelifesciences.com). The membranes were incubated with the specified primary antibodies at 4°C overnight. Primary antibodies include anti-phospho-p53 (1∶1,000, Cell Signaling, Danvers, MA, http://www.cellsignal.com, #9281), anti-p53 (1∶400, Santa Cruz Biotechnology Inc., Santa Cruz, CA, http://www.scbt.com, #sc-6243) and anti-GAPDH (1∶5,000, Ambion, Austin, TX, Ambion, Austin, TX, www.ambion.com, #4300). After washing with TBST, the membrane was respectively incubated with secondary antibody ECL anti-rabbit IgG or anti-mouse IgG (GE Healthcare Life Sciences) for 1 h at room temperature. Signals were detected with ECL plus western blotting detection system (GE Healthcare Life Sciences).

### RNA isolation and reverse transcription-polymerase chain reaction

Total RNA was isolated using the RNeasy Mini Kit incorporating DNase I as suggested by the manufacturer. Reverse transcription was carried out as follows: 2 µg of RNA and random primers (3 µg/µl) were incubated for 3 min in 70°C and cooled on ice. Next, the master mix was added, consisting of following components: 5.0 µl of 5x reaction buffer (Promega), 0.5 µl of (25 mM) dNTP, 0.1 µl of M-MLV (Moloney murine leukemia virus) reverse transcriptase (200 U/µl; USB) and 9.4 µl of dH_2_O. The reaction was stopped at 65°C for 10 min after 1 h incubation at 42°C. The cDNA was used as template for real-time PCR in order to confirm the Illumina array-derived data.

### Real-time PCR

Real-time polymerase chain reaction (PCR) was carried out on the Applied Biosystems 7900 instrument, in 96-Well Optical Reaction Plates (Applied Biosystems, Foster City, CA, United States). The following program was applied: stage 1∶ 50°C for 2 min, stage 2∶ 95°C for 10 min, stage 3∶ 95°C for 15 s and 60°C for 1 min, for 40 cycles and, stage 4∶ 95°C for 15 s, 60°C for 15 s and 95°C for 15 s. Additional dissociation curves of the products were created. The final reaction volume of 20 µl consisted of 10 µl of SYBR Green PCR Master Mix (Applied Biosystems), 2.5 µM of each primer (3 µl), and 7 µl of cDNA (1∶8 dilution). Each gene was analyzed in triplicate. One biological replicate was used for HFF1 cells transduced with four factors (4F)-OSKM, GFP and polybrene-treated cells. Confirmation of the Illumina array results for HFF1 cells after 24, 48 and 72 h post-transduction with viruses was investigated. Relative mRNA levels were calculated using the comparative Ct method [81] and presented as a percentage of the biological controls (untreated HFF1 cells). mRNA levels of *GAPDH* was used as control for normalisation.

### Detection of cellular senescence

For the staining of senescent cells, the Senescence beta-Galactosidase Staining Kit (Cell Signaling, Danvers, MA, USA, www.cellsignal.com) was used following the manufacturer's protocol. Briefly, 24, 48 or 72 h post-transduction, HFF1 cells were washed, fixed and incubated overnight with the staining solution. Finally, the nuclei were counter stained using DAPI/PBS (100 ng/ml) for 12 min at room temperature. Nuclei and senescent cells (blue cytoplasmatic beta-galactosidase staining) were visualized and images were acquired using the confocal microscope LSM 510 Meta (Zeiss). Processing of images was carried out using AxioVision V4.6.3.0 (Zeiss) and Adobe Photoshop CS version 8.0 (Adobe, Munich, Germany, www.adobe.com) software. Quantification of nuclei was performed using the ImageJ software (version 1.43), whereas senescent cells were manually counted for three snap shots of each duplicate.

## Supporting Information

Figure S1 **Additional GO categories enriched in regulated transcripts.** Data is represented as in Figure 3. Panel A. GO categories related to apoptosis, cell proliferation and cell cycle are shown. Panel B. GO categories related to morphological changes and aging are shown.
(TIF)

Figure S2 **Time series clusters.** Transcripts regulated at any time point (24 h, 48 h or 72 h; 2636 transcripts; padj <0.05) were divided into nine clusters by fuzzy-c-means clustering. Each trace is color coded according to the membership value of the gene to the respective cluster. The number of genes in the 0.5 alpha-core of the cluster is detailed in Data S4.
(TIF)

Figure S3 **Regulated transcript enrichment within KEGG focal adhesion pathway overlaid with transcriptional changes of the differentially expressed genes in the pathway.** Each rectangle is divided into segments representing changes at 24, 48, 72 hours and iPS state compared to untreated HFF1.
(TIF)

Figure S4 **EMT suppression signature.** Panel A. Recovery Test for EMT gene ranking: This distribution shows the performance of the EMT ranking and may also be used to estimate the quality of the seed. Ten percent of the EMT genes (seed) are repeatedly taken out and the position of this left out group in the rank is determined. A good performance results in a clear tendency to show high frequencies for top positions (left side). A random seed would result in a uniform distribution (flat histogram). Statistics: p-value: p<10–20. The Null-Hypothesis for this test is that the relative probability to be in the most left bin is not larger in comparison with the relative probability in the rest of the histogram. The p-value is obtained from the cumulative binomial distribution. Panel B. EMT Suppression: Spearman's rank correlation rho of the genes of an EMT enriched genome wide list were rank correlated (Spearman rank correlation) with the significant most down regulated genes at each time point (starting with the most down regulated with the rank). A high rho corresponds to a down-regulation of positive EMT genes. The results indicate a progressive down regulation of positive EMT associated genes during the reprogramming process.
(TIF)

Figure S5 **Quantitative real-time PCR validation.** Quantitative real-time PCR analysis of the genes involved in innate immune response to viruses (e.g. CCL5, IRF7, STAT2, TRIM5, DDX58, MX2, IL12A, EIF2AK2, ISG20; panel A) and induction of apoptosis (e.g. IL19, NGEF; STAT1, CASP1; panel B) in HFF1 cells transduced with OSKM in comparison with HFF1 cells at 24 h, 48 h and 72 h post-transduction. Expression values were normalized over the expression of GAPDH and presented as relative changes compared to HFF1 cells. Blue bars: QRT-PCR. Red bars: array values.
(TIF)

Figure S6 **Transduction efficiency of retroviral transduction and nucleofection.** GFP vector was transduced into HFF1 cells using retroviral transduction or nucleofection procedure. Percentages of GFP positive cells were measured by flow cytometry 24 h post-transduction.
(TIF)

Data S1 **6179 transcripts regulated at any time point or in ES/iPS.** Column descriptions: pid (Illumina probe identifier); profile.id (text string describing its regulation in the following groups with respect to HFF1: 24 h, 48 h, 72 h, iPS, ES; 1 = regulated (padj <0.05), 0 = not regulated); t.24 h.logFC, t48 h.logFC, t72 h.logFC, diPS.logFC, dES.logFC (log2 fold changes with respect to HFF1; log2FC is set to zero if padj<0.05 and log2FC <log2(1.5); entrez (Entrez Gene ID), symbol (Entrez Gene Symbol); gene.name (Entrez Gene Name). Filename: Suppl_Data_S1.txt.
(TXT)

Data S2 **Pluripotency- and fibroblast-associated transcripts and potential pluripotent cell markers.** Filename: Suppl_Data_S2.xls.
(XLS)

Data S3 **GO categories enriched in regulated transcripts at 24 h, 48 h, 72 h post-transduction and in iPS and ES cells compared to HFF1 cells.** Filename: Suppl_Data_S3.xls.
(XLS)

Data S4 **Transcripts in Fuzzy c-means clusters and KEGG pathways enriched per cluster.** Cluster #7 was the one described in the main text. Filename: Suppl_Data_S4.xls.
(XLS)
