# Supplementary figures and images for: Molecular Insights into Reprogramming-Initiation Events Mediated by the OSKM Gene Regulatory Network
Source: PLoS One. 2011 Aug 31;6(8):e24351. doi: 10.1371/journal.pone.0024351 (PMC3164204; doi:10.1371/journal.pone.0024351)

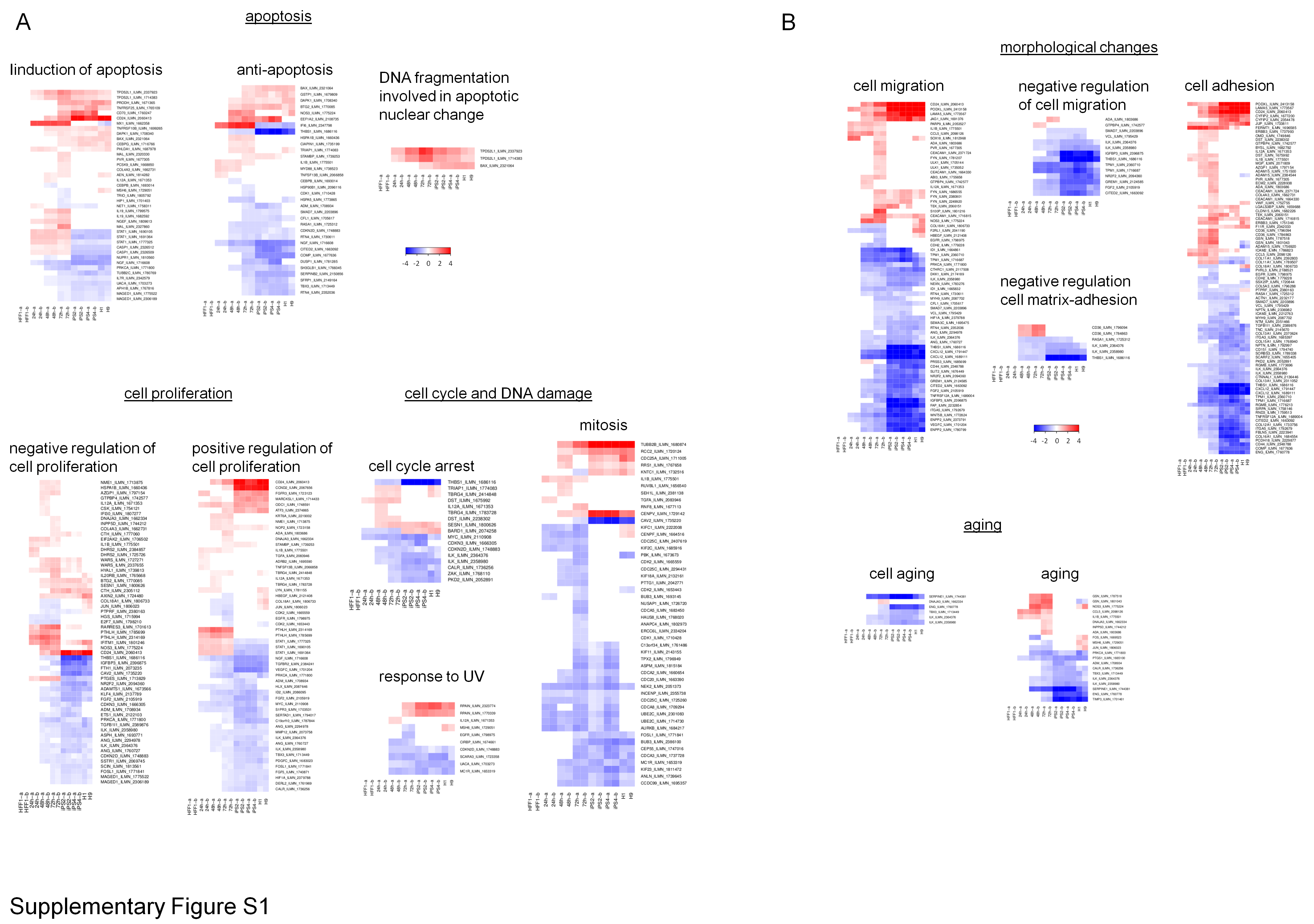

Supplement: Figure S1 — Additional GO categories enriched in regulated transcripts. Data is represented as in Figure 3. Panel A. GO categories related to apoptosis, cell proliferation and cell cycle are shown. Panel B. GO categories related to morphological changes and aging are shown. (TIF) [file pone.0024351.s001.tif]

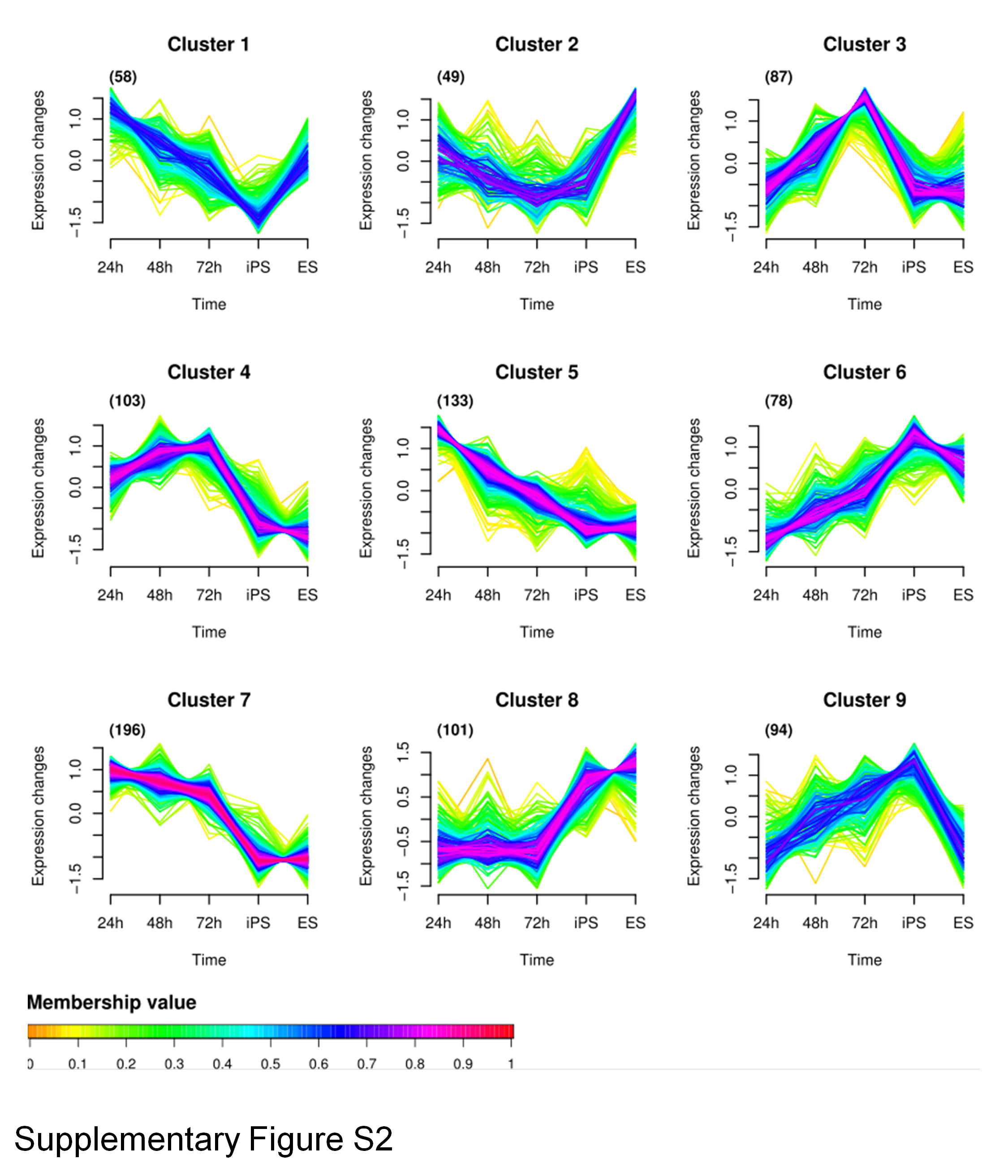

Supplement: Figure S2 — Time series clusters. Transcripts regulated at any time point (24 h, 48 h or 72 h; 2636 transcripts; padj <0.05) were divided into nine clusters by fuzzy-c-means clustering. Each trace is color coded according to the membership value of the gene to the respective cluster. The number of genes in the 0.5 alpha-core of the cluster is detailed in Data S4. (TIF) [file pone.0024351.s002.tif]

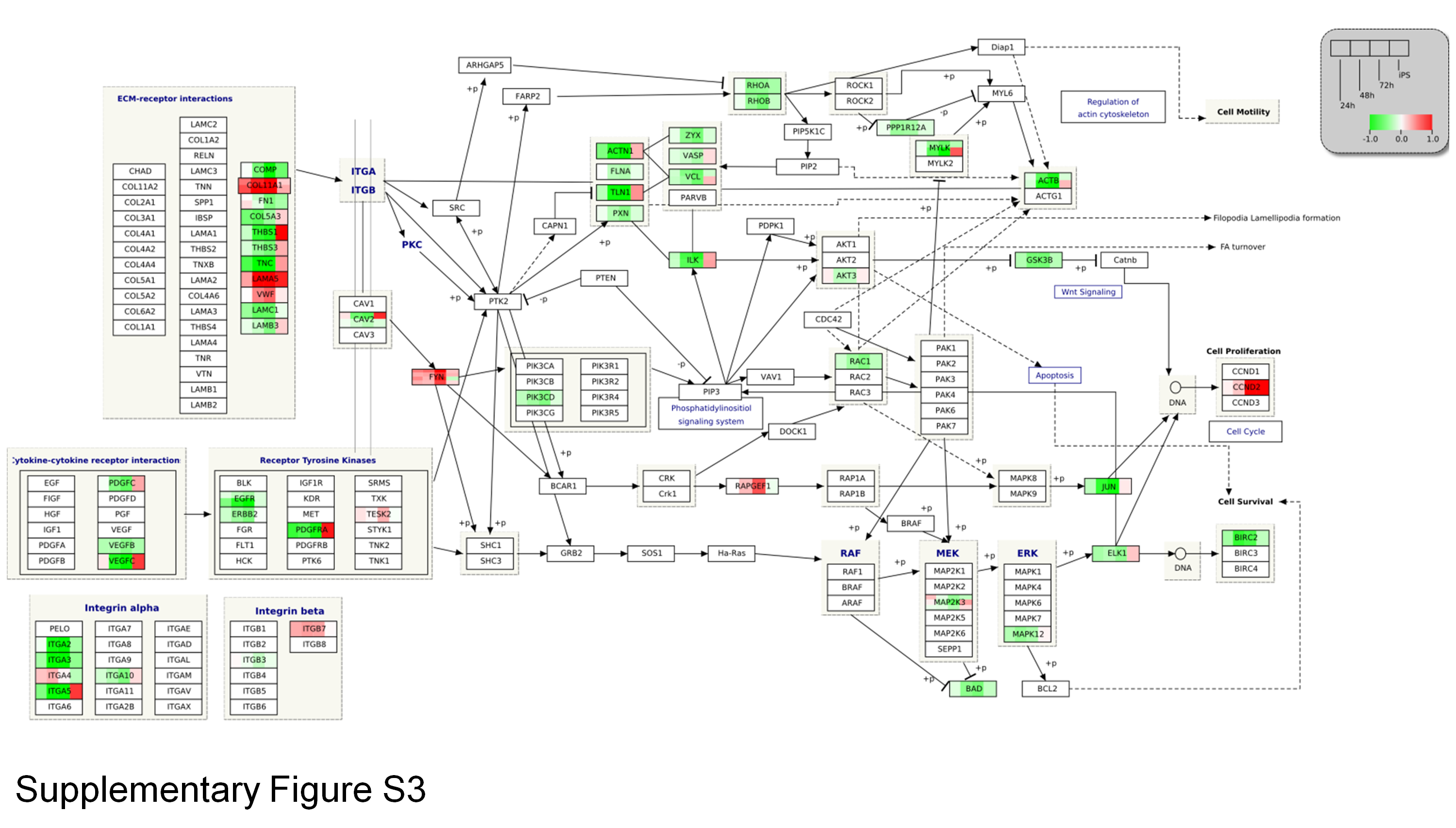

Supplement: Figure S3 — Regulated transcript enrichment within KEGG focal adhesion pathway overlaid with transcriptional changes of the differentially expressed genes in the pathway. Each rectangle is divided into segments representing changes at 24, 48, 72 hours and iPS state compared to untreated HFF1. (TIF) [file pone.0024351.s003.tif]

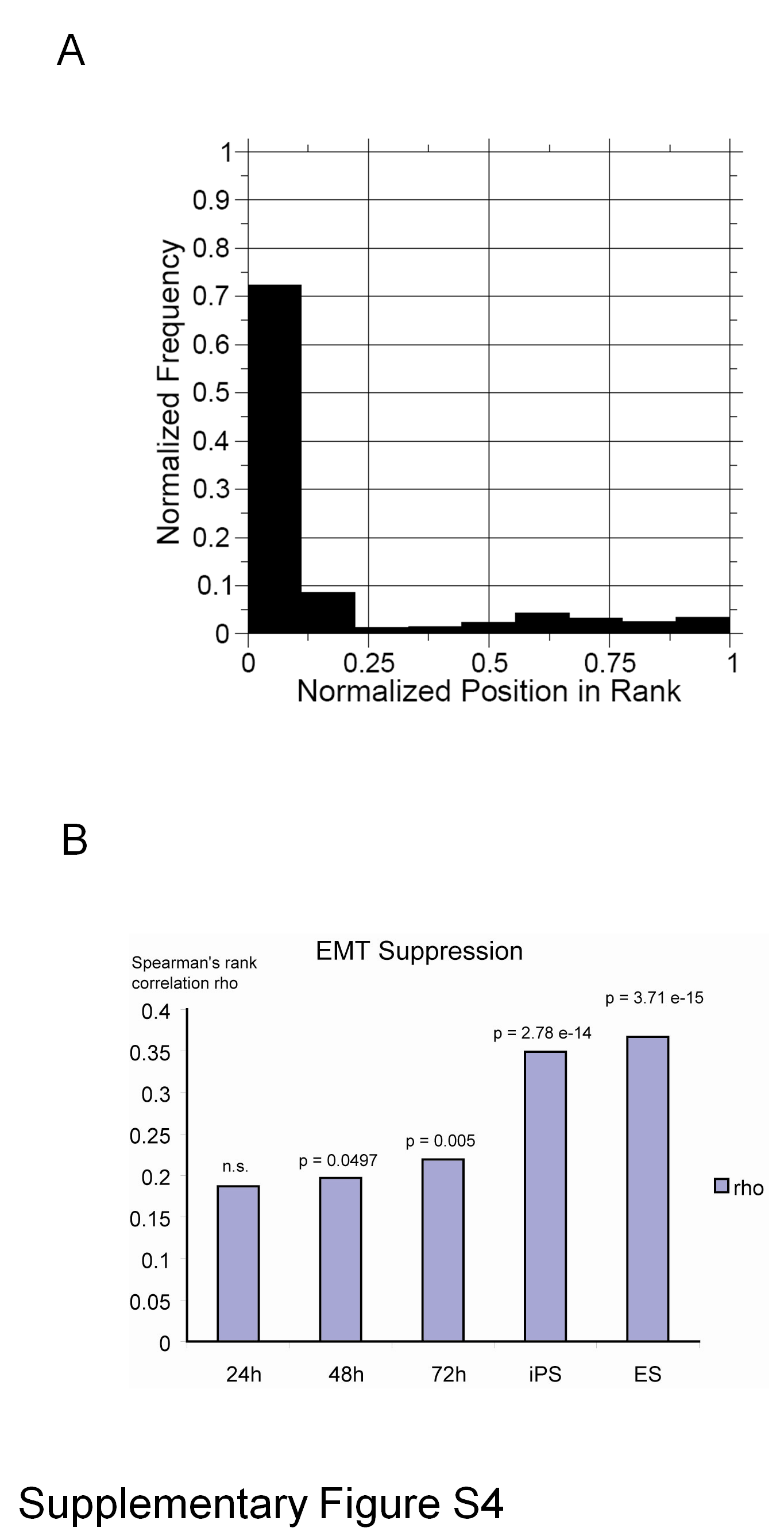

Supplement: Figure S4 — EMT suppression signature. Panel A. Recovery Test for EMT gene ranking: This distribution shows the performance of the EMT ranking and may also be used to estimate the quality of the seed. Ten percent of the EMT genes (seed) are repeatedly taken out and the position of this left out group in the rank is determined. A good performance results in a clear tendency to show high frequencies for top positions (left side). A random seed would result in a uniform distribution (flat histogram). Statistics: p-value: p<10–20. The Null-Hypothesis for this test is that the relative probability to be in the most left bin is not larger in comparison with the relative probability in the rest of the histogram. The p-value is obtained from the cumulative binomial distribution. Panel B. EMT Suppression: Spearman's rank correlation rho of the genes of an EMT enriched genome wide list were rank correlated (Spearman rank correlation) with the significant most down regulated genes at each time point (starting with the most down regulated with the rank). A high rho corresponds to a down-regulation of positive EMT genes. The results indicate a progressive down regulation of positive EMT associated genes during the reprogramming process. (TIF) [file pone.0024351.s004.tif]

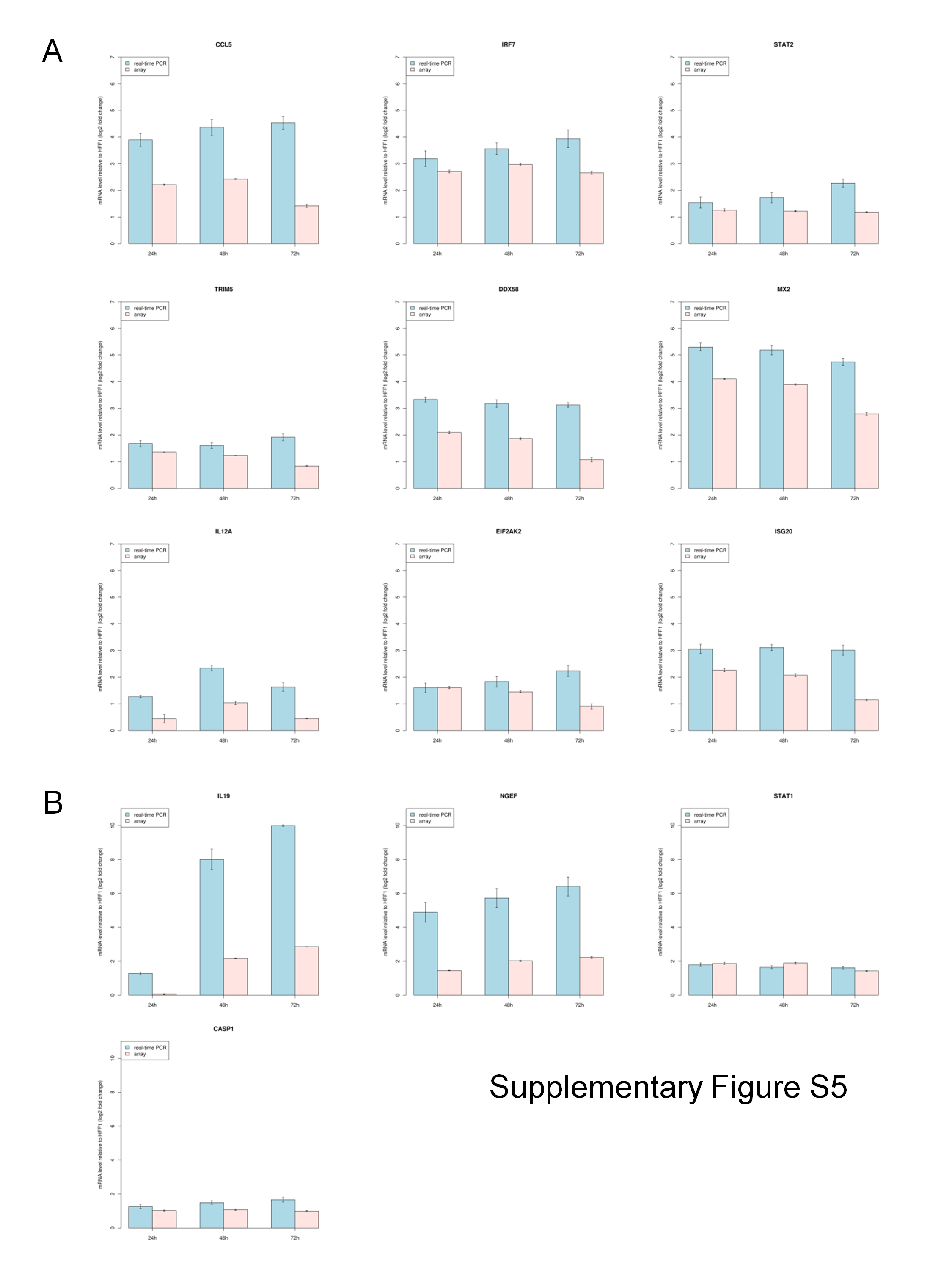

Supplement: Figure S5 — Quantitative real-time PCR validation. Quantitative real-time PCR analysis of the genes involved in innate immune response to viruses (e.g. CCL5, IRF7, STAT2, TRIM5, DDX58, MX2, IL12A, EIF2AK2, ISG20; panel A) and induction of apoptosis (e.g. IL19, NGEF; STAT1, CASP1; panel B) in HFF1 cells transduced with OSKM in comparison with HFF1 cells at 24 h, 48 h and 72 h post-transduction. Expression values were normalized over the expression of GAPDH and presented as relative changes compared to HFF1 cells. Blue bars: QRT-PCR. Red bars: array values. (TIF) [file pone.0024351.s005.tif]

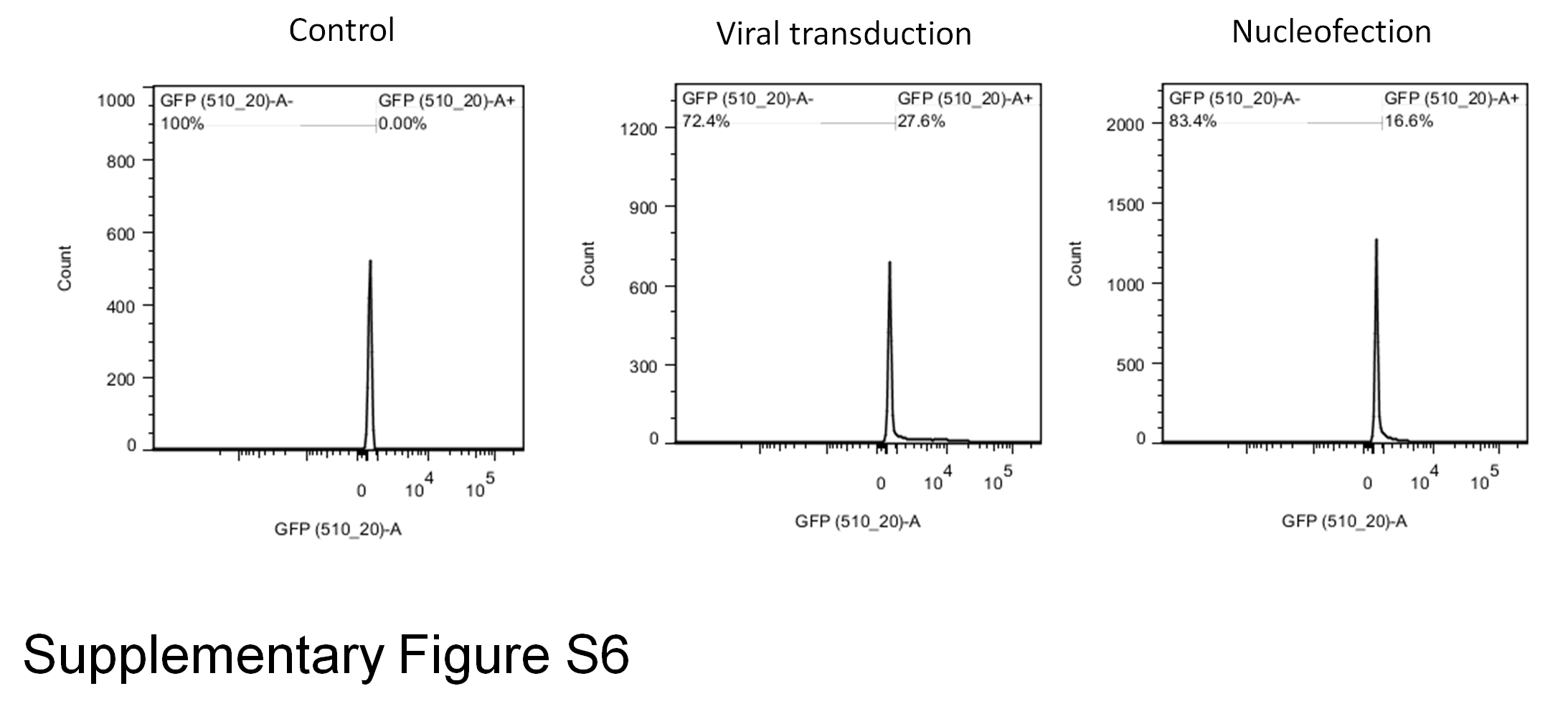

Supplement: Figure S6 — Transduction efficiency of retroviral transduction and nucleofection. GFP vector was transduced into HFF1 cells using retroviral transduction or nucleofection procedure. Percentages of GFP positive cells were measured by flow cytometry 24 h post-transduction. (TIF) [file pone.0024351.s006.tif]
